# Bnip3 interacts with vimentin, an intermediate filament protein, and regulates autophagy of hepatic stellate cells

**DOI:** 10.18632/aging.202211

**Published:** 2020-12-03

**Authors:** Jie Liu, Yuyu Xie, Zhangbo Cui, Tian Xia, Lu Wan, Haifeng Zhou, Peng Zhang, Yijie Zhang, Fei Guan, Wenqi Liu, Chunwei Shi

**Affiliations:** 1 Department of Pathogen Biology, School of Basic Medicine, Tongji Medical College, Huazhong University of Science and Technology, Wuhan, PR China; 2 Department of Surgery, Wuhan Third Hospital, Wuhan, PR China; 3 Department of Hospital Infection Management, Wuhan Children’s Hospital (Wuhan Maternal and Child Healthcare Hospital), Tongji Medical College, Huazhong University of Science and Technology, Wuhan, PR China; 4 Department of Pathophysiology, School of Basic Medicine, Tongji Medical College, Huazhong University of Science and Technology, Wuhan, PR China; 5 Department of Immunology, School of Basic Medicine, Tongji Medical College, Huazhong University of Science and Technology, Wuhan, PR China; 6 Department of Integrated Traditional Chinese and Western Medicine, Union Hospital, Tongji Medical College, Huazhong University of Science and Technology, Wuhan, PR China

**Keywords:** hepatic stellate cells, autophagy, Hif-1, Bnip3, vimentin

## Abstract

Bnip3, which is regulated by Hif-1 in cells under oxygen deprivation, is a death related protein associated with autophagy and apoptosis. Hif-1 was reported to regulate autophagy to activate hepatic stellate cells (HSCs), while the specific molecular mechanism is vague. The possible mechanism of Hif-1 regulating autophagy of HSCs via Bnip3 was explored in this study. Bnip3 was detected in fibrotic liver tissues from humans and mice. Hif-1 was inhibited by chemical inhibitor and Bnip3 was detected in activated HSCs. The co-localization of Bnip3 and LC3B was captured by confocal microscopy and autophagic flow was assessed in *Bnip3* siRNA transfected cells. Bnip3 interacted proteins were screened with mass spectrometry. The interaction of Bnip3 and vimentin was detected with co-immunoprecipitation and confocal microscopy. The results showed that Bnip3 was increased in fibrotic liver tissues and activated HSCs. Hif-1 inhibition suppressed Bnip3 expression in activated HSCs. Bnip3 was partially co-localized with autophagosomes and Bnip3 inhibition suppessed autophagy in activated HSCs. Bnip3 interacted with vimentin and Bnip3 expression was inhibited as vimentin was inhibited in activated HSCs. Conclusively, this study indicated that Bnip3 promoted autophagy and activation of HSCs, via interacting with vimentin, an intermediate filament protein with highly abundant expression in HSCs.

## INTRODUCTION

Liver fibrosis is characterized by excessive scar formation due to overproduction and deposition of the extracellular matrix (ECM) [1]. Hepatic stellate cells (HSCs) were identified as the key cellular source of extracellular matrix in the liver. Upon acute or chronic liver damage, HSCs transdifferentiate from quiescent, lipid droplet-containing cells toward myofibroblast-like cells, which were characterized by a decreased number of lipid droplets, increased proliferation, increased expression of vimentin and α-smooth muscle actin (α-SMA), enhanced synthesis of ECM [2].

Autophagy, by which cells degrade and metabolize their own constituents, is an evolutionary conserved fundamental cellular process. Bilayer membrane components in cells encapsulate damaged organelles or proteins of cytoplasm, to form vesicles denominated autophagosomes. Autophagosomes fuse with lysosomes to form autophagolysosomes, which degrade the contents to adapt cellular metabolic requirement or remove damaged organelles [3–5]. Recent studies have determined that, during the activation of HSCs upon liver damage, different autophagic mechanisms including classical macro-autophagy, mitophagy, lipophagy, and multiple organelles including mitochondria, lipid droplets and endoplasmic reticulum, are involved [6–9], illustrating that HSC activation is a complex and sophisticated process, which remains unclear.

At present, many genes involved in autophagy have been identified. Among them, Bnip3 (Bcl-2/adenovirus E1B 19-kDa interacting protein), was determined to be associated with autophagy and apoptosis. Bnip3 belongs to the Bcl-2 family and contains a single Bcl-2 homology domain (BH3). In different kinds of cells, Bnip3 is distributed differently, mainly localized in the nuclear membrane, mitochondria and endoplasmic reticulum [10, 11]. Previous studies reported that Bnip3 was a pro-apoptotic protein and mediated apoptosis in caspase-dependent or caspase-independent manner [11, 12]. Recently, it was found that Bnip3 is a death-related protein with a wide range of biological effects and Bnip3 also promotes autophagy through various mechanisms in different cells. Bnip3, localized in the outer membrane of mitochondria, interacts with LC3 (microtubule-associated protein light chain 3) to form the complex of mitochondrial Bnip3 and LC3, leading to the induction of mitophagy [11, 13]. Autophagy-related protein Beclin-1 also has BH3 region, which will be released to induce autophagy, as Bnip3 competes with Beclin-1 via its BH3 domain to bind with Bcl-2 or Bcl-Xl [14, 15]. Bnip3 was reported to bind with Rheb, a regulatory protein upstream in mTOR (mammalian rapamycin target protein) signaling pathway, resulting in the inhibition of mTORC1 and induction of autophagy [16].

Hypoxia inducible factor-1 (Hif-1) is an important transcriptional factor that mediates cellular responses to hypoxia and stressors like infection and inflammation. It is a heterodimer consisting of an α-subunit Hif-1α and β-subunit Hif-1β. Hif-1α is highly regulated by micro-environmental oxygenation, whereas Hif-1β is constitutively expressed. Our previous researches have determined that Hif-1 regulates autophagy to activate HSCs [17]. The expression of autophagy related genes in hypoxia-stimulated HSCs was analyzed by whole-genome expression microarray and a differentially expressed autophagy related gene, *Bnip3*, which was also reported to be Hif-1 target gene, was screened out [18]. In this study, we used human HSC cell line LX-2 and culture-activated primary HSCs from mice as cell models, to further explore the possible mechanism of Bnip3 regulating autophagy and activation in hepatic stellate cells, thus to reveal the detailed mechanism of hepatic fibrosis.

## RESULTS

### The expression of Bnip3 was increased in fibrotic liver tissues from clinical patients and *Schistosoma japonicum* infected mice

**Figure 1:**
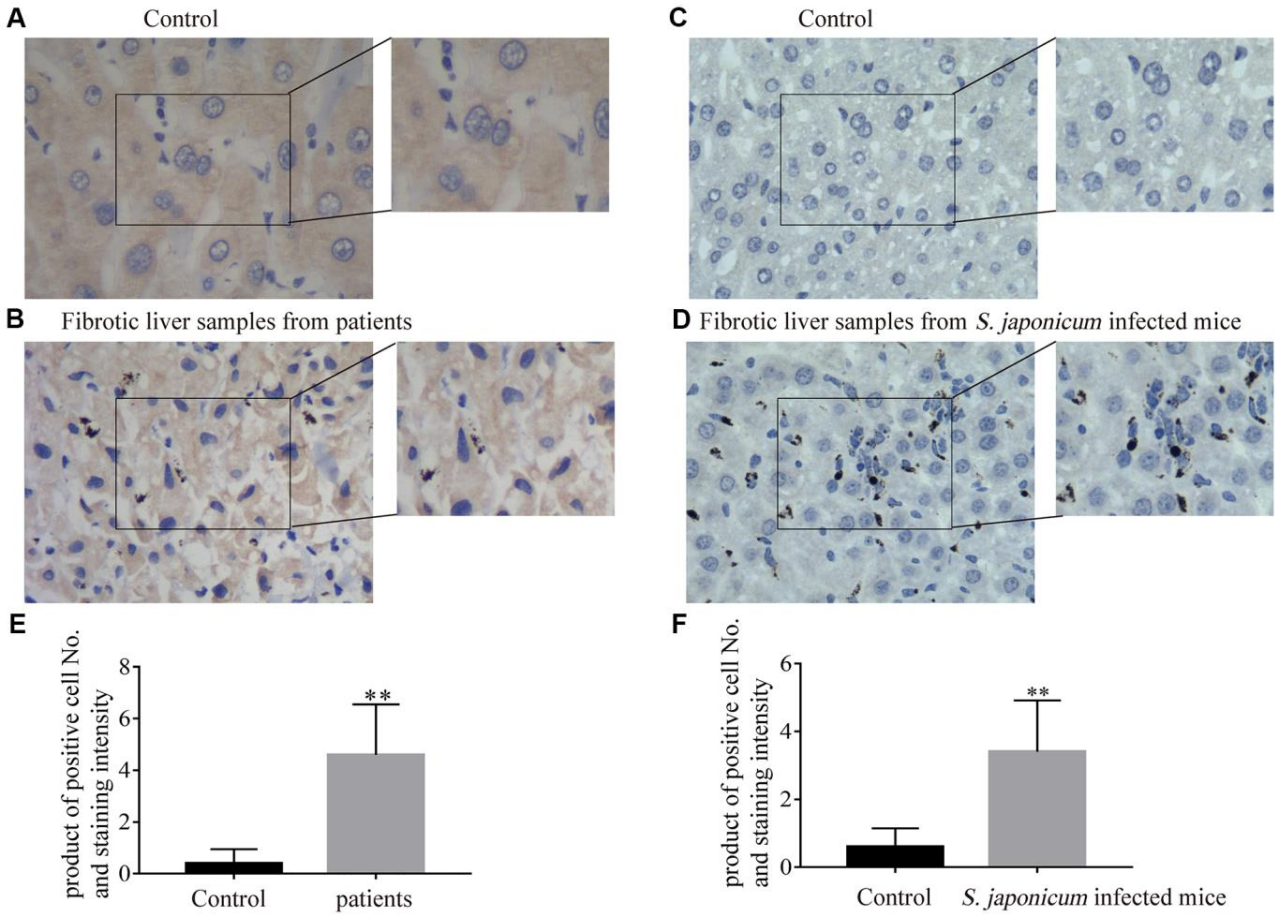
**The expression of Bnip3 was increased in fibrotic liver tissues from clinical patients and *Schistosoma japonicum* infected mice.** Expression of Bnip3 was identified in liver biopsy specimens from clinical patients, and mice infected with *S. japonicum* by immunohistochemistry. (**A, B**) Human samples from liver fibrosis were collected for Bnip3 detection by immunohistochemistry and liver samples of para-hemangioma from patients, were used as negative control. (**A**) Liver samples of para-hemangioma from patients (n=8). (**B**) Fibrotic liver samples from patients (n=8). Scale bar, 20 μm. (**C, D**) BALB/c female mice, 6–8 weeks old, were percutaneously infected with 25 cercariae of *Schistosoma japonicum* through the shaved abdomen, sacrificed at 8 weeks post-infection, and samples of liver were collected. (**C**) Non-infected liver samples from mice (n=4) and (**D**) fibrotic liver samples from *S. japonicum* infected mice (n=4). Scale bar, 20 μm. Representative images were shown. Positive cells were counted and the scores were multiplied with staining intensity. Products were introduced into SPSS 19.0 to analyze statistical difference of Bnip3 expression in normal liver tissues and fibrotic liver tissues. (**E**) Bnip3 in liver tissues from patients; (**F**) Bnip3 in liver tissues from *S. japonicum* infected mice. **P < 0.01.

Expression of Bnip3 was firstly detected in fibrotic liver samples from clinical patients. Compared with non-fibrotic liver samples (Figure 1A), fibrotic liver tissues had obviously higher expression of Bnip3 (Figure 1B, 1E). Bnip3 was also detected in fibrotic liver tissues of mice infected with *S. japonicum*, which was recognized as an animal model of infective fibrosis [17–20]. It was shown that Bnip3 increased in fibrotic liver tissue from infected mice, as compared with normal liver tissues from non-infected mice (Figure 1C, 1D, 1F). From the morphological characteristics, it was suggested that Bnip3 positive cells were mainly non-parenchymal hepatic cells (Figure 1B, 1D). Collectively, Bnip3 was increasingly expressed in fibrotic liver tissues.

### The activation of hepatic stellate cells induced Bnip3 expression and its cytoplasmic translocation in vitro

**Figure 2:**
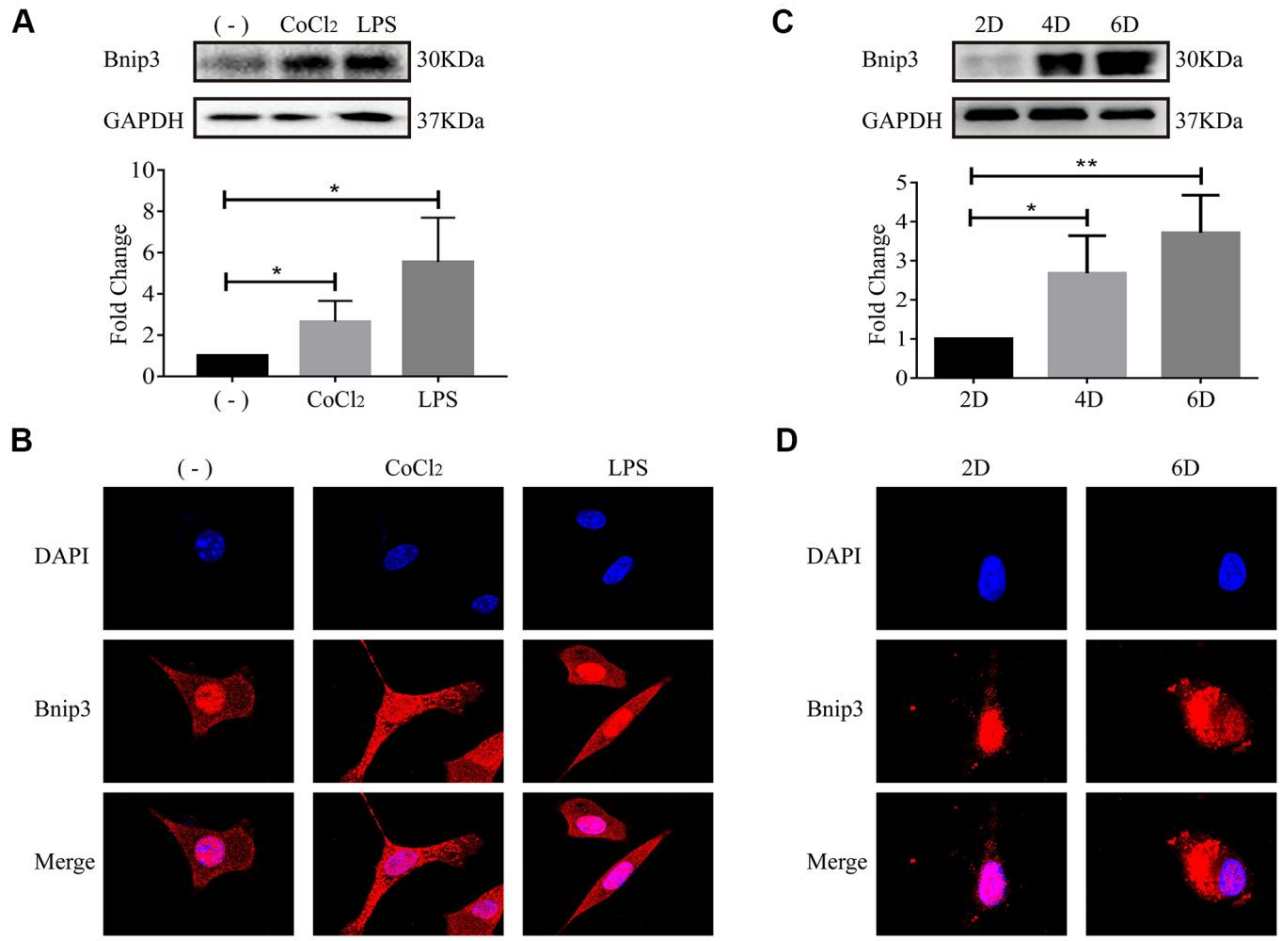
**The activation of hepatic stellate cells induced Bnip3 expression and its cytoplasmic translocation in vitro.** LX-2 cells were treated with 100 μM CoCl_2_ or 2 μg/ml LPS for 8 h. (**A**) Cells were collected at indicated time and cell lysates were subjected to detect Bnip3 with Western blot. Densitometric analysis for Western blot was performed and data were expressed as mean ± SD, *P < 0.05. (**B**) Immunofluorescence assay was performed to detect Bnip3 (Cy3) in CoCl_2_- or LPS-treated LX-2 cells. Culture-activated primary HSCs from mice were cultured up to 2 days, 4 days or 6 days. (**C**) Cells were collected at indicated time and cell lysates were subjected to detect Bnip3 with Western blot. Densitometric analysis for Western blot was performed and data were expressed as mean ± SD, *P < 0.05, **P < 0.01. (**D**) Immunofluorescence assay was performed to detect Bnip3 (Cy3) and images were captured by confocal microscope.

We previously reported that human hepatic stellate cell line, LX-2, was activated upon hypoxia or LPS stimulation, along with the increase of Hif-1 [17–20]. Increased expression of Bnip3 was also confirmed in both hypoxia or LPS-stimulated LX-2 cells (Figure 2A). In immunofluorescence staining, it was observed that Bnip3 was localized in nucleus in non-stimulated control LX-2 cells, while in hypoxia and LPS-stimulated cells, Bnip3 displayed obvious cytoplasmic localization (Figure 2B). Consistent results were observed in primary HSCs isolated from mice. We have previously determined that in in-vitro culture, HSCs were naturally activated [19]. The increased expression of Bnip3 in activated primary HSCs was also confirmed by Western blot, which indicated that Bnip3 increasingly expressed as the primary HSCs were activated in-vitro cultured up to 4 or 6 days, as compared with cells cultured up to 2 days (Figure 2C). Nuclear Bnip3 expression was also observed in primary HSCs cultured up to day 2, while in cells cultured up to day 6, Bnip3 was mainly expressed in cytoplasm (Figure 2D). The results all above demonstrated that Bnip3 expressed increasingly and translocated from nucleus to cytoplasm during HSC activation.

### Autophagy occurred when hepatic stellate cells were activated, along with increase of Hif-1

**Figure 3:**
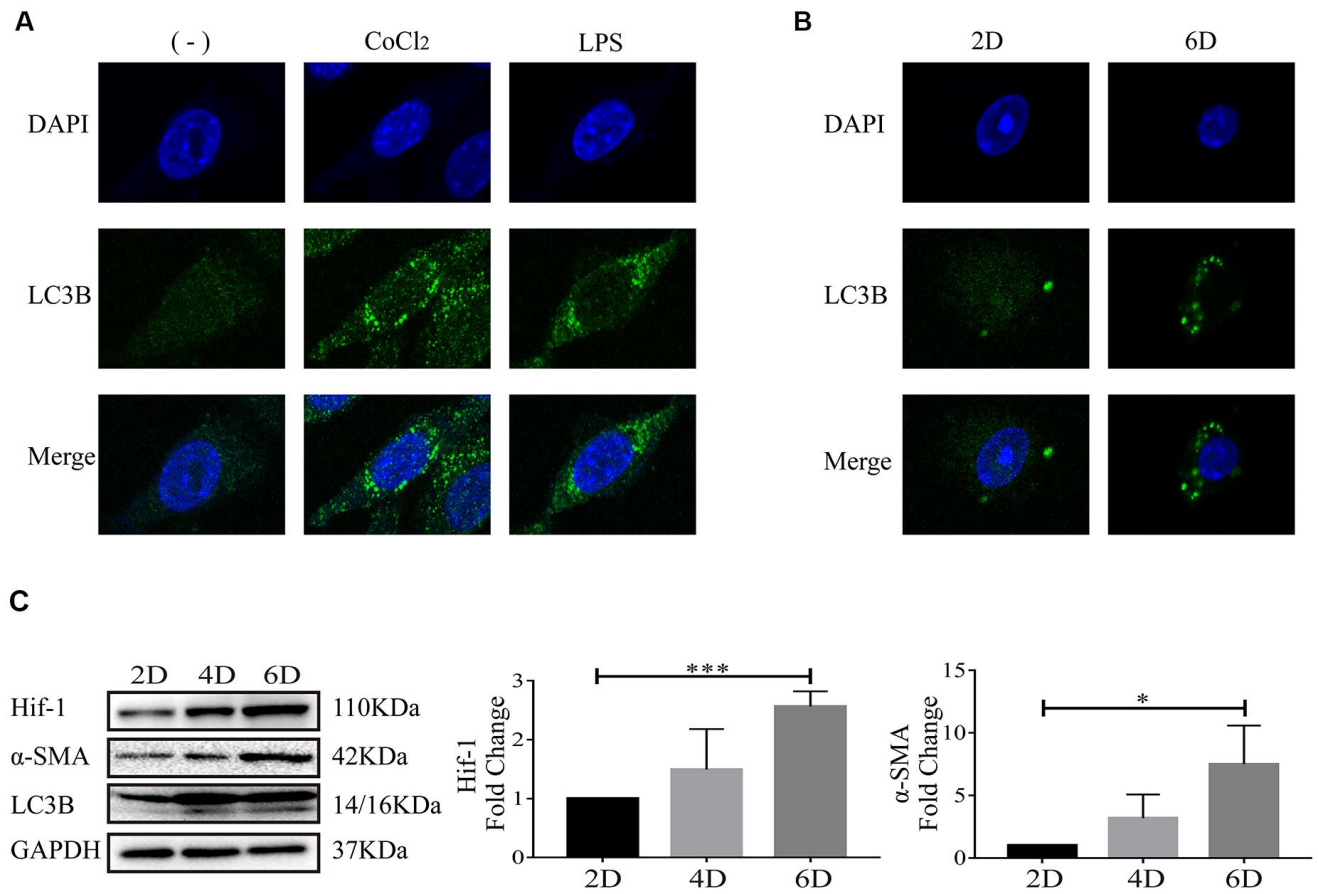
**Autophagy occurred when hepatic stellate cells were activated.** (**A**) LX-2 cells were treated with 100 μM CoCl_2_ or 2 μg/ml LPS for 8 h. (**B**) Culture-activated primary HSCs from mice were cultured up to 2 days or 6 days. Immunofluorescence assay was performed to detect LC3B (FITC) in CoCl_2_- or LPS-treated LX-2 cells (**A**) and culture-activated primary HSCs from mice cultured up to 2 days or 6 days (**B**). Images were captured by confocal microscope. (**C**) Culture-activated primary HSCs from mice were cultured up to 2 days, 4 days or 6 days. Cells were collected at indicated time and cell lysates were subjected to detect Hif-1α, α-SMA and LC3B with Western blot. Densitometric analysis for Western blot was performed and data were expressed as mean ± SD, *P < 0.05, ***P < 0.001.

We have previously reported that Hif-1 regulates autophagy to activate hepatic stellate cells, using LX-2 cells, human HSC cell line, as the cell model [17]. LC3 (microtubule-associated protein light chain 3), a mammalian homolog of yeast Atg8, is a widely recognized protein for monitoring autophagic activity. There are three isoforms of LC3 in mammalian cells including LC3A, LC3B and LC3C, among which, LC3B is a widely used autophagy marker [21]. After synthesis, LC3 is cleaved to generate 16-kDa cytosolic LC3 and 14-kDa lipidated LC3. 14-kDa lipidated LC3 is the membrane component involved in formation of autophagosomes and autophagolysosomes [22, 23]. In this study, formation of autophagosomes indicated by LC3B punctate particles and 14-kDa lipidated LC3B were used to detect autophagy activity. In LX-2 cells, it was observed that CoCl_2_ or LPS stimulation resulted in apparent accumulation of LC3B (Figure 3A), which is consistent with our previous study [17]. Molecular markers of autophagy, including LC3B punctate particles (Figure 3B) and 14-kDa lipidated LC3B (Figure 3C) were respectively detected with confocal microscopy and Western blot in primary HSCs. Expression of Hif-1α, α-SMA, molecular marker of HSC activation, was also detected (Figure 3C). As the primary HSCs were cultured in vitro, with the extending of in-vitro culture time, increase of α-SMA indicated the activation of primary HSCs and increase of Hif-1α demonstrated the activation of Hif-1 signaling cascade in primary HSCs (Figure 3C). Increase of 14-kDa lipidated LC3B and accumulation of LC3B punctate particles in primary HSCs cultured in vitro up to day 6, indicated that autophagic degradation enhanced along with the activation of primary HSCs (Figure 3B, 3C). Bnip3 was previously reported to be associated with both autophagy and apoptosis. Apoptosis was detected in hypoxia/LPS-stimulated LX-2 cells and the results indicated that apoptosis did not occur in our in-vitro culture system (Supplementary Figure 1). The above results demonstrated the enhanced autophagy in activated HSCs, with the activation of Hif-1 signaling.

### Inhibition of Hif-1α suppressed the increased expression of Bnip3 in hepatic stellate cells

**Figure 4:**
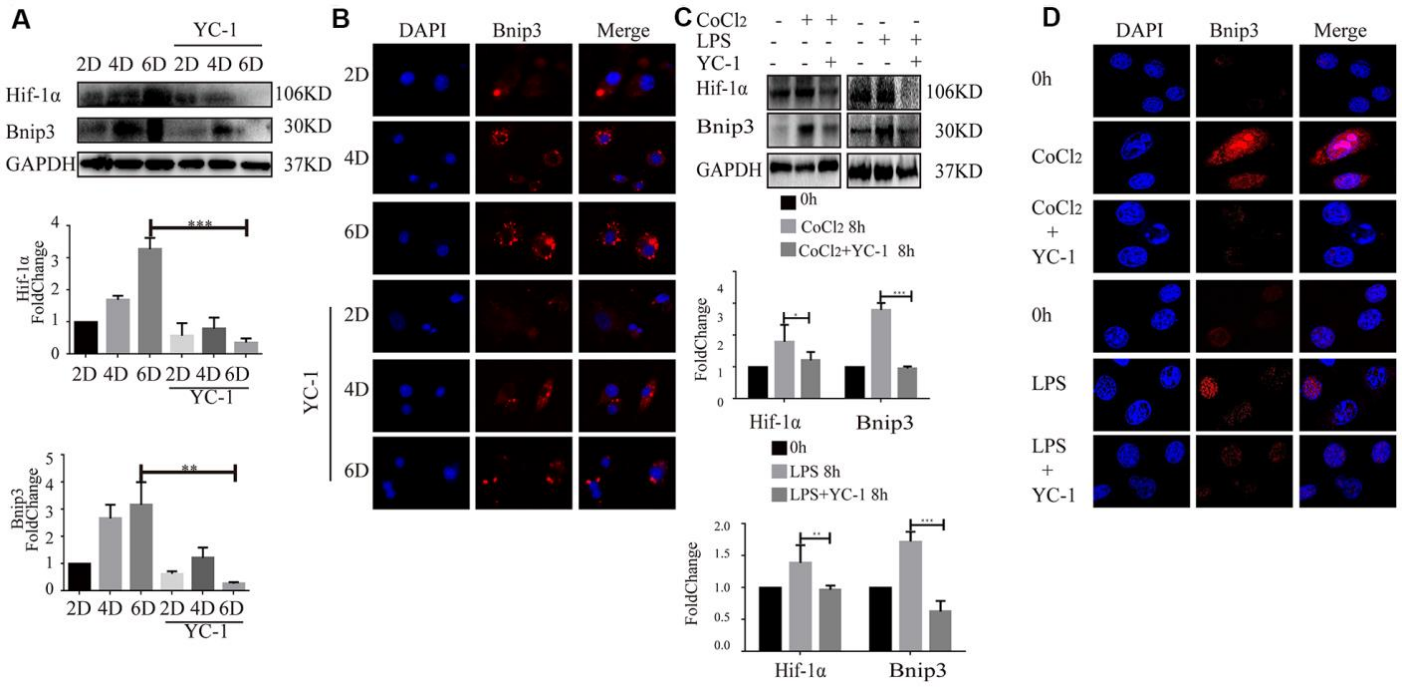
**Inhibition of Hif-1 suppressed increased expression of Bnip3 in activated hepatic stellate cells.** Culture-activated primary HSCs from mice were cultured up to 2 days, 4 days or 6 days and Hif-1 chemical inhibitor YC-1 (50 μM) was added as cells were cultivated to 2 days. (**A**) Cell lysates were subjected to detect Hif-1α and Bnip3 with Western blot. Densitometric analysis was performed and data were expressed as mean ± SD, *P < 0.05, **P < 0.01, ***P < 0.001. (**B**) Immunofluorescence assay was performed to detect Bnip3 (Cy3) by confocal microscopy. (**C**) LX-2 cells were stimulated by 100 μM CoCl_2_ or 2 μg/ml LPS either alone or after YC-1 pre-treatment (50 μM). Cell lysates were subjected to detect Hif-1α and Bnip3 with Western blot. Densitometric analysis was performed and data were expressed as mean ± SD, *P < 0.05, **P < 0.01, ***P < 0.001. (**D**) Immunofluorescence assay was performed to detect Bnip3 (Cy3) by confocal microscopy.

Bnip3 was previously reported to be regulated by Hif-1 in cells upon hypoxia [24], however, the relationship between Hif-1 and Bnip3 was still unclear in hepatic stellate cells. YC-1, chemical inhibitor of Hif-1α, was used to inhibit Hif-1α expression. In primary HSCs, as Hif-1α expression was efficiently inhibited by YC-1, Bnip3 expression was suppressed in in-vitro cultured and activated primary HSCs (Figure 4A, 4B). Similar results were observed in LX-2 cells. LX-2 cells were pretreated with YC-1 and then stimulated with CoCl_2_ or LPS. The results from Western blot (Figure 4C) and immunofluorescence staining (Figure 4D) showed that the inhibition of Hif-1 expression led to the suppress of Bnip3 in hypoxia or LPS activated LX-2 cells. Collectively, these results indicated that Bnip3 expression was regulated by Hif-1 during the activation of hepatic stellate cells.

### Bnip3 was partially co-localized with autophagosomes in activated HSCs and inhibition of Bnip3 led to the blockage of the autophagic flow and activation of HSCs

**Figure 5:**
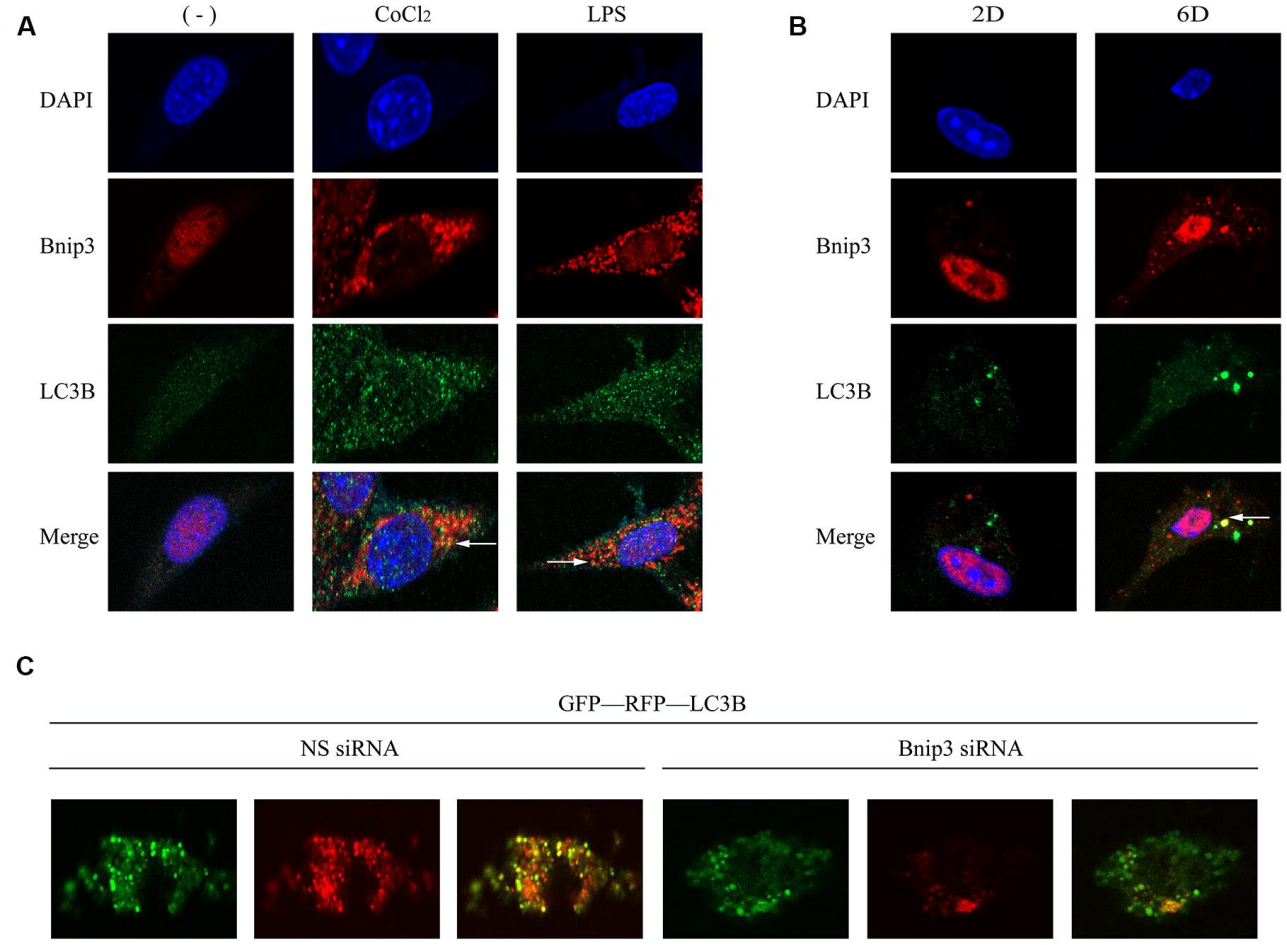
**Bnip3 was partially co-localized with autophagosomes in activated HSCs and inhibition of Bnip3 led to the blockage of the autophagic flow.** LX-2 cells were treated with 100 μM CoCl_2_ or 2 μg/ml LPS for 8 h. Culture-activated primary HSCs from mice were cultured up to 2 days or 6 days. Immunofluorescence assay was performed to detect Bnip3 (Cy3) and LC3B (FITC) in LX-2 cells (**A**) and culture-activated primary HSCs from mice (**B**) by confocal microscopy. (**C**) Primary HSCs were isolated from mice and seeded in coverslips. Cells were transfected with specific siRNA targeting *Bnip3* as cells were cultivated up to day 2 and GFP-RFP-LC3B plasmid was transfected into cells 24h later. Cells were fixed at day 6 and images were captured by confocal microscope to observe the formation of autophagosomes.

Bnip3 and LC3B were co-stained to observe whether Bnip3 was co-localized with autophagosomes in activated HSCs. It was shown that in both hypoxia/LPS-stimulated LX-2 cells (Figure 5A) and culture-activated primary HSCs from mice (Figure 5B), Bnip3 was partially co-localized with LC3B punctate particles. Autophagic flux was further detected in primary HSCs transfected with *Bnip3* siRNA. Primary cells were firstly transfected with *Bnip3* siRNA at day 2 and GFP-RFP-LC3B plasmid was transfected 24h later. Cells were further cultured until day 6 and formation of autophagosomes was observed. In control cells transfected with non-specific siRNA, punctate particles of both GFP-LC3B (autophagosomes) and RFP-LC3B (autophagolysosomes) were formed, when merged, non-degraded autophagic bodies (yellow dots) and degraded autophagic bodies (red dots) were clearly visible, which indicated the functional autophagy in control cells (Figure 5C). However, in *Bnip3* interfered primary HSCs, signals of both GFP-LC3B (autophagosomes) and RFP-LC3B (autophagolysosomes) were attenuated. Especially, the signals of RFP-LC3B (autophagolysosomes) were obviously weaker than the signals of GFP-LC3B (autophagosomes) (Figure 5C), which indicated the blockage of autophagic flow due to *Bnip3* interference.

Molecular marker of HSC activation, α-SMA, was detected in CoCl_2_/LPS-stimulated LX-2 cells (Supplementary Figure 2A, 2B) and primary HSCs (Supplementary Figure 2C) transfected with *Bnip3*-specific siRNA. It was shown that *Bnip3* interference inhibited increase of Bnip3 and α-SMA. Collectively, the results above demonstrated that Bnip3 played an important role in the autophagy and activation of HSCs.

### Bnip3 interacted with vimentin in activated hepatic stellate cells

**Figure 6:**
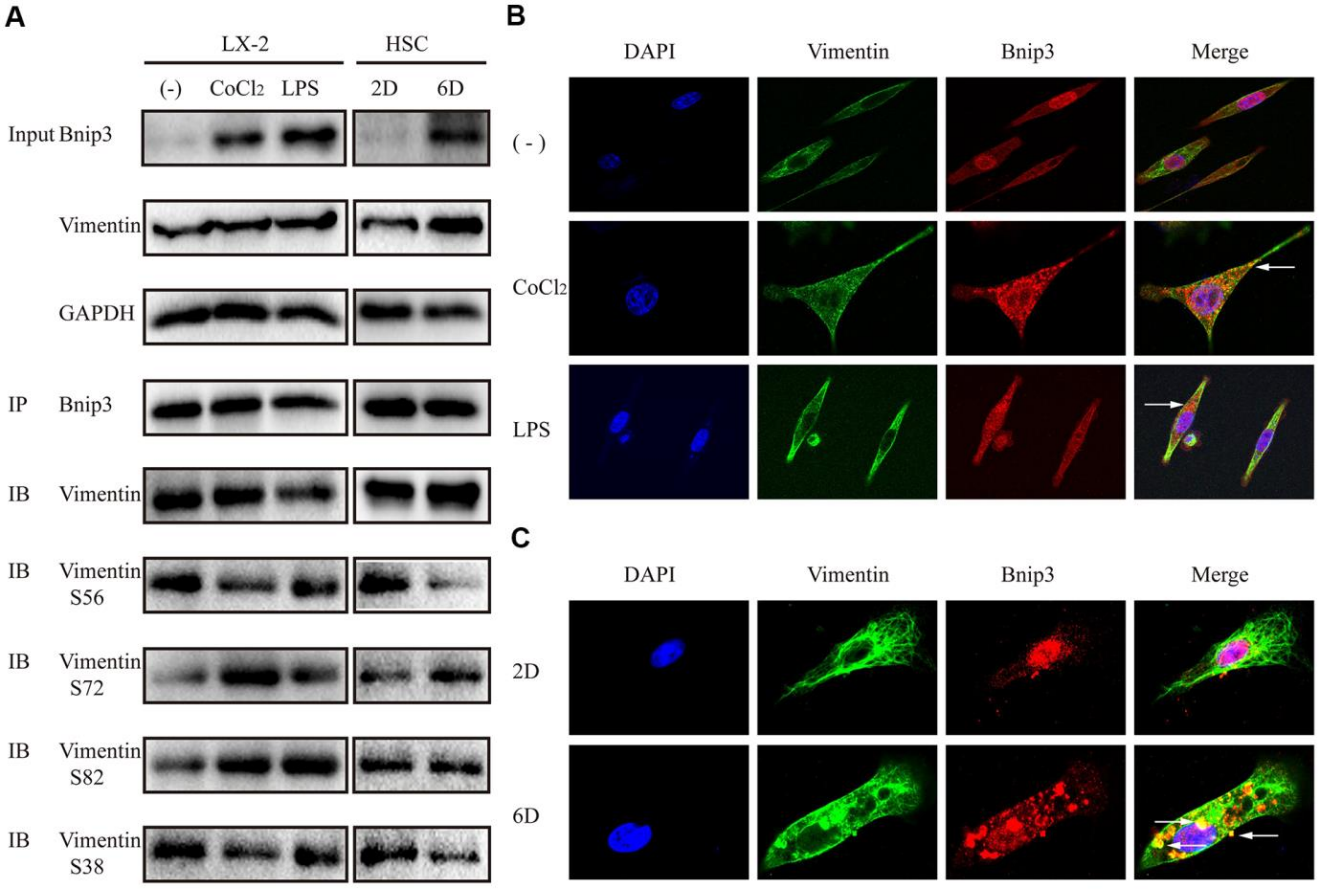
**Bnip3 interacted with vimentin in activated hepatic stellate cells.** LX-2 cells were treated with 100 μM CoCl_2_ or 2 μg/ml LPS for 8 h. Culture-activated primary HSCs from mice were cultured up to 2 days or 6 days. (**A**) 300μg cell lysates of LX-2 cells and 150μg cell lysates of primary HSCs from mice were collected and immunoprecipitated with anti-Bnip3, followed by immunoblotting with anti-vimentin, anti-phosphorylated vimentin S56, S72, S82 and S38. (**B, C**) Immunofluorescence assay was performed to detect Bnip3 (Cy3) and vimentin (FITC) in LX-2 cells (**B**) and culture-activated primary HSCs from mice (**C**) by confocal microscopy.

Proteins interacted with Bnip3 were further screened in hypoxia-stimulated LX-2 cells with mass spectrometry (Supplementary Tables 1, 2). It was reported from mass spectrometry that Bnip3 might have interaction with a kind of intermediate filament (IF) protein, vimentin, which has highly abundant expression in HSCs [25]. Antibody specific to Bnip3 was used as the bait to detect vimentin with co-immunoprecipitation and the interaction of Bnip3 and vimentin was confirmed in both hypoxia/LPS stimulated LX-2 cells and culture-activated primary HSCs (Figure 6A). As vimentin has multiple phosphorylation sites at serine residues, phosphorylated vimentin at serine residue 56, serine residue 72, serine residue 82 and serine residue 38, was also detected in Bnip3 interacted complex. In hypoxia/LPS stimulated LX-2 cells, as compared with control cells, phosphorylations at serine residue 72 and 82 were enhanced, while phosphorylations at serine residue 56 and 38 were reduced (Figure 6A). In primary HSCs cultured up to day 6, as compared with cells cultured up to day 2, phosphorylation at serine residue 72 was enhanced, phosphorylations at serine residue 56 and 38 were reduced, while phosphorylation at serine residue 82 showed no change (Figure 6A). Apparent co-localization of Bnip3 and vimentin was captured by confocal microscopy in both hypoxia/LPS stimulated LX-2 cells (Figure 6B) and primary HSCs cultured up to day 6 (Figure 6C). Withaferin A, a chemical inhibitor targeting vimentin [26], which induces aggregation and fragmentation of vimentin, was used to inhibit vimentin activity in LX-2 cells. It was shown that inhibition of vimentin re-organization with Withaferin A inhibited increased expression of Bnip3 in activated LX-2 cells. Autophagy marker LC3B was also detected and 14-kDa lipidated LC3B was inhibited with Withaferin A treatment in LX-2 cells (Supplementary Figure 3). Collectively, the above results demonstrated that in activated HSCs, Bnip3 regulated the autophagy and activation of HSCs, possibly via interacting with vimentin, a kind of IF protein with highly abundant expression in HSCs.

## DISCUSSION

Activation of hepatic stellate cells is the key mechanism during the development of liver fibrosis. We have previously determined that Hif-1, hypoxia inducible factor-1, affects the activation of hepatic stellate cells by regulating autophagy [17, 18, 20]. The expression of autophagy related genes was analyzed by whole genome expression chip in LX-2 cells stimulated by chemical hypoxia and Bnip3, which is a multifunctional protein involved in regulating apoptosis and autophagy, was screened out [18].

Hif-1 is an important regulator mediating cellular responses to hypoxia and stressors like infection and inflammation [27]. LPS, lipopolysaccharide, which is a component of the cell wall of Gram-negative bacteria, can reach the liver through the portal vein circulation, leading to the liver inflammation. LPS/TLR4 pathway in HSCs is related to the development of liver fibrosis, which regulates the expression of pro-inflammatory cytokines and controls cell survival [28, 29]. In both our previous work [17–19] and current work, we determined that LPS stimulation induced increased expression of Hif-1 and Hif-1 also increasingly expressed in activated primary HSCs from mice, which indicated an important regulatory role of Hif-1 in HSC activation. As a transcriptional regulator, Hif-1 enters into the nucleus to affect the expression of a series of its target genes, including *Bnip3*, encoding Bcl-2/adenovirus E1B 19-kDa protein interacting protein [30, 31]. *Bnip3* is a typical hypoxia responsive gene and its promoter region contains 2 or 3 HREs in different species including rat, mice and humans [24]. In this work, it was observed that during HSC activation, the expression of Hif-1α and Bnip3 was increased, while the expression of Bnip3 was inhibited in the activated cells, as Hif-1 was inhibited, which suggested that the expression of Bnip3 was regulated by Hif-1 in HSC activation.

It has been reported that Bnip3 regulates autophagy through a variety of mechanisms in different cells, including mechanisms of mitophagy, dissociation of Beclin-1 from Bcl-2, or interaction with upstream regulatory protein Rheb in mTOR signal pathway [13–16]. Proteins interacted with Bnip3 in HSCs were analyzed with mass spectrum and the possible interaction of Bnip3 and vimentin, the major intermediate filament protein in HSCs was predicted. The intermediate filament (IF) proteins are cytoskeletal proteins that form 10-nm filaments in cells, which comprise the cytoskeleton, along with microtubules and actin microfilaments, and provide the cells with shape and structural integrity. The main IF proteins existing in HSCs are class III IF proteins, including desmin, GFAP (glial fibrillary acidic protein) and vimentin. It was reported that expression of desmin and vimentin increased during HSC activation, while GFAP was down-regulated during HSC transdifferentiation [25]. Increase of vimentin is a marker for HSC activation and assembly of large number of IF bundles during HSC transdifferentiation requires vimentin [25]. Recently, it was reported that vimentin was involved in regulation of autophagosome and lysosome trafficking [32].

The phosphorylation modification of multiple serine residues of vimentin was further confirmed in Bnip3-vimentin complex. In hypoxia/LPS stimulated LX-2 cells and activated primary HSCs from mice, phosphorylation modification of the main serine residues of vimentin, including serine residue 72, 56, 38, was consistent, while phosphorylation at serine residue 82 was not consistent. Vimentin has a highly complex phosphorylation pattern with different sites and cellular status [33]. In this study, the mechanism and function of phosphorylation at different serine residues of vimentin in Bnip3-vimentin complex were not well defined, however, this result further confirmed that Bnip3 interacted with vimentin in activated HSCs.

The function of cytoskeletal proteins in autophagy has been recognized for a long time, while the detailed mechanisms about their cooperative effect are still puzzling. It was reported that vimentin plays a physiological role in autophagosome and lysosome positioning, through regulating the activity of mTORC1 [32]. In this study, it was observed that in activated HSCs, Bnip3 was partially co-localized with LC3B, but the two molecules did not overlap completely. Bnip3 is a multifunctional protein in cells, which is located in different sub-cellular structures such as the outer membrane of mitochondria, endoplasmic reticulum and nuclear membrane. The partial co-localization of Bnip3 with LC3B and the involvement of Bnip3 in HSC autophagy and activation, suggested us that cellular distribution of Bnip3 in HSCs, including localization in mitochondria, lipid droplets, endoplasmic reticulum or nucleus, should be firstly clarified in the future work. Based on that, we supposed that Bnip3 interacted with vimentin to re-position Bnip3-anchored organelles, to promote autophagy of HSCs.

Nuclear expression of Bnip3 was determined in tumors or some diseases [34–36]. It was suggested that nuclear Bnip3 acts as the transcriptional regulator and binds with promoters of various genes related with cell survival or cell death. When Bnip3 translocates from nucleus to cytoplasm, Bnip3 associates with organelles such as mitochondria and endoplasmic reticulum, to promote apoptosis or autophagy [34–36]. In our study, localization of Bnip3 in nucleus was also observed in quiescent HSCs, including non-stimulating LX-2 cells and quiescent primary HSCs. As HSCs were activated, Bnip3 expressed increasingly and translocated from nucleus to cytoplasm. A chemical inhibitor targeting vimentin, Withaferin A, which induces aggregation and fragmentation of vimentin [26], was used to inhibit vimentin activity in LX-2 cells. It was shown that inhibition of vimentin re-organization with Withaferin A inhibited increased expression of Bnip3 in activated LX-2 cells, which indicated that activities of vimentin is indispensable to Bnip3 expression in activated HSCs and also mechanisms regulated by Bnip3 including the autophagy and activation of HSCs. Whether the translocation of Bnip3 from nucleus to cytoplasm depends on intermediate filaments composed with vimentin, is worthy of in-depth study.

Conclusively, in current study, it was determined that Bnip3, regulated by Hif-1α, promoted autophagy during HSC activation. In activated HSCs, Bnip3 regulated the autophagy and activation of HSCs, possibly via interacting with vimentin, a kind of intermediate filament protein with highly abundant expression in HSCs.

## MATERIALS AND METHODS

### Ethics statement and human studies

Animal experiments were approved by the Committee on Animal Research of Tongji Medical College (TMC), Huazhong University of Science and Technology, Hubei Province, PR China. Human studies were approved by the institutional review board of TMC. All patients provided written informed consent. Eight cases of liver tissues from patients with liver fibrosis diagnosed by liver biopsy and normal liver tissues surrounding hepatic hemangioma excised from surgical operation were collected.

### Animal study

BALB/c female mice, 8 weeks old, were randomly divided into two groups: the infected group and the control group. *S. japonicum* cercariae were shed from the snails. Each anaesthetized mouse in the infected group was percutaneously infected with 25 cercariae through the shaved abdomen. The mice were sacrificed at 8 weeks post-infection, and samples of liver were collected [17].

### Isolation of primary cells and cell culture

Primary hepatic stellate cells were isolated from healthy BALB/c mouse by enzymatic digestion and Percoll density gradient centrifugation [19]. Primary HSCs were cultured in DMEM (Hyclone, USA) supplemented with 16% inactivated fetal bovine serum (Gibco, USA) as appropriate. LX-2, human HSC line, was grown in DMEM supplemented with 10% inactivated fetal bovine serum (Sciencell, USA) as appropriate. The antibiotics penicillin G (100 U/mL) and streptomycin sulfate (100 μg/mL) were added.

### Transfection, cell stimulation and chemical inhibitors

Cells were transfected with 50nM siRNA specific to *Bnip3* or non-specific siRNA using Ribo FECTTM CP transfection kit following manual instruction (RiboBio, China). Lipofectamine 2000 (Invitrogen, USA) was used to transfect GFP-RFP-LC3B plasmid into cells. Cells were stimulated by 100μM CoCl_2_ (Sigma, C8661, USA) or 2μg/ml LPS (Sigma, L-2880, USA). YC-1 (MCE, 170632-47-0, USA) was used as Hif-1 chemical inhibitor and Withaferin A (MCE, HY-N2065, USA) was used as chemical inhibitor of vimentin re-organization.

### Antibodies

Primary antibodies used for Western blot, immunohistochemistry and immunocytochemistry are listed as followed: Hif-1α (79233, Cell signaling, USA), α-SMA (ab32575, Abcam, USA), Bnip3 (ab10433, Abcam, USA; ab38621, Abcam, USA), LC3B (83506, cell signaling, USA; 12741, cell signaling, USA), vimentin (ServiceBio, GB12192, China), phosphor-vimentin Ser56 (ab217673, Abcam, USA), phosphor-vimentin Ser38 (ab52942, Abcam, USA), phosphor-vimentin Ser72 (ab52944, Abcam, USA), phosphor-vimentin Ser82 (ab52943, Abcam, USA), caspase 3 (ab197202, Abcam, USA), cytochrome C (sc-13156, Santa Cruz, USA), GAPDH (GB12002, Servicebio, China).

### Western blot

Protein concentration from cell lysates was valued using BCA Protein Assay Kit (P0011, Beyotime, China). Protein samples were separated by SDS/PAGE and transferred onto PVDF membrane (Millipore, USA). After blocking in 5% BSA, membranes were incubated with primary antibodies and then corresponding secondary antibodies. Immunoreactive bands were tested with SuperSignal TM ELISA Femto Maximum Sensitivity Substrate (37075, Thermo, USA).

### Immunohistochemistry and immunocytochemistry

The formalin-fixed and paraffin-embedded liver tissues were cut into 4-μm sections and the expression of Bnip3 was determined in tissue of normal and fibrotic livers. Cells seeded on coverslips were fixed in 4% paraformaldehyde, permeabilized with 0.1% Triton X-100, and counter-stained with antibodies against Bnip3, LC3B or vimentin. The coverslips were mounted onto slides in anti-fade mounting medium (H-1000, Vectorlabs, USA) and fluorescent images were captured using confocal microscopy (FV3000, Olympus, Japan).

### Immunoprecipitation and mass spectrometry

Cells were lysed in 4° C pre-cooled RIPA buffer and 150-300 μg of cell lysate was incubated with 1 μg Bnip3 monoclonal antibody at 4° C overnight with continuous agitation. Protein A+G Agarose (P2012, Beyotime, China) was added and incubated for additional 2 hours at 4° C. The beads were washed and precipitated proteins were eluted by boiling the beads in 2×SDS-PAGE sample buffer for 5 minutes. The samples were analyzed by Western blot or LC-MS/MS-based analysis.

### Flow cytometry analysis of apoptosis

Cells were stained with Annexin V-FITC/PI detection kit according to the manual instruction (AD-10, Dojindo, Japan). Apoptotic cells were detected using flow cytometry (AFC2, Life Technologies, Singapore) and apoptosis was calculated with percentage of Q2 cells (late apoptosis) and Q4 cells (early apoptosis).

### Statistical analysis

All data are expressed as mean±SD. Differences between experimental and control groups were assessed by one-way ANOVA or unpaired, two-sided Mann–Whitney U-test using SPSS 19.0, *P < 0.05, **P < 0.01, ***P < 0.001.

## Supplementary Material

Supplementary Figures

Supplementary Table 1

Supplementary Table 2
