# Supplementary material for: Bnip3 interacts with vimentin, an intermediate filament protein, and regulates autophagy of hepatic stellate cells
Source: Aging (Albany NY). 2020 Dec 3;13(1):957–72. doi: 10.18632/aging.202211 (PMC7834981; doi:10.18632/aging.202211)
Supplement: Supplementary Figures [file aging-13-202211-s001.pdf]

SUPPLEMENTARY FIGURES

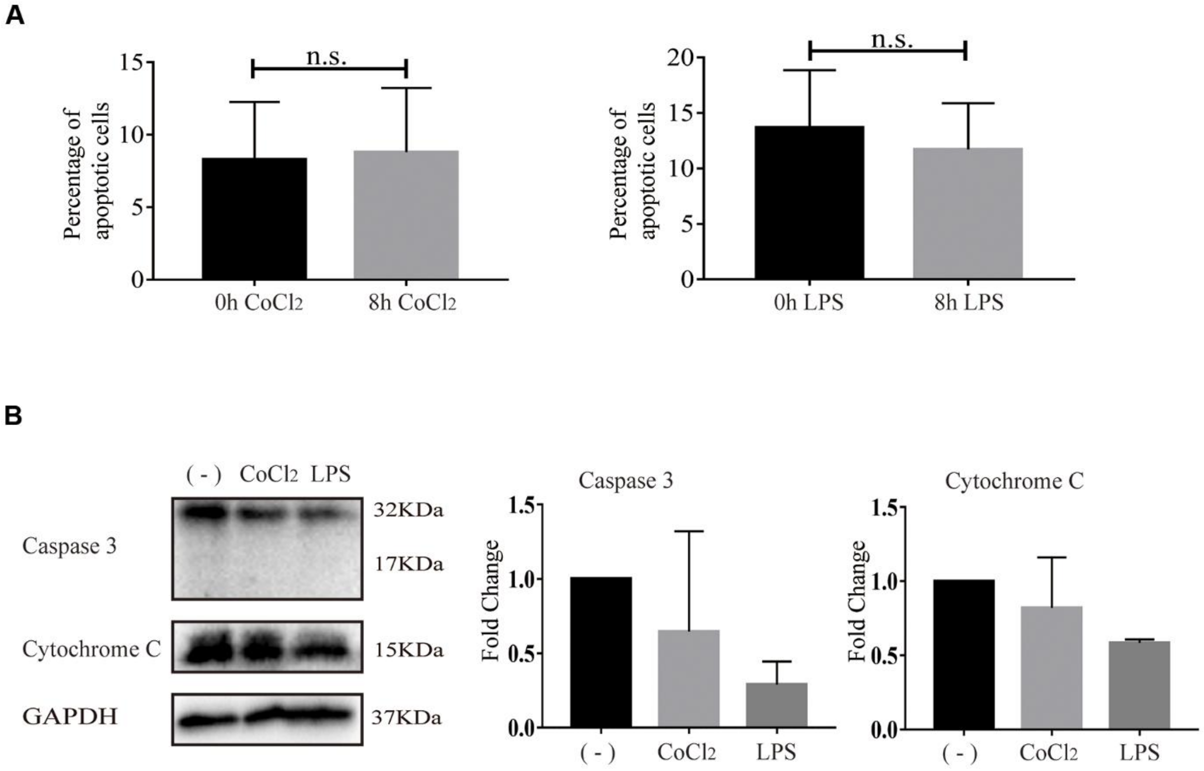

Supplementary Figure 1. Apoptosis did not occur during activation of hepatic stellate cells. LX-2 cells were treated with 100 μM $CoCl_2$ or 2 μg/ml LPS for 8 h. (**A**) Cells were collected and stained with Annexin V-FITC/PI apoptosis double staining kit. Apoptosis was analyzed with FACS. Annexin V+/PI- cells were recorded as early apoptotic cells and Annexin V+/PI+ cells were recorded as late apoptotic cells to calculate the percentage of apoptotic cells. Densitometric analysis was performed and data were expressed as mean ± SD. (**B**) Cell lysates were subjected to detect caspase 3 and cytochrome C with Western blot. Densitometric analysis was performed and data were expressed as mean ± SD. n.s.: not significant.

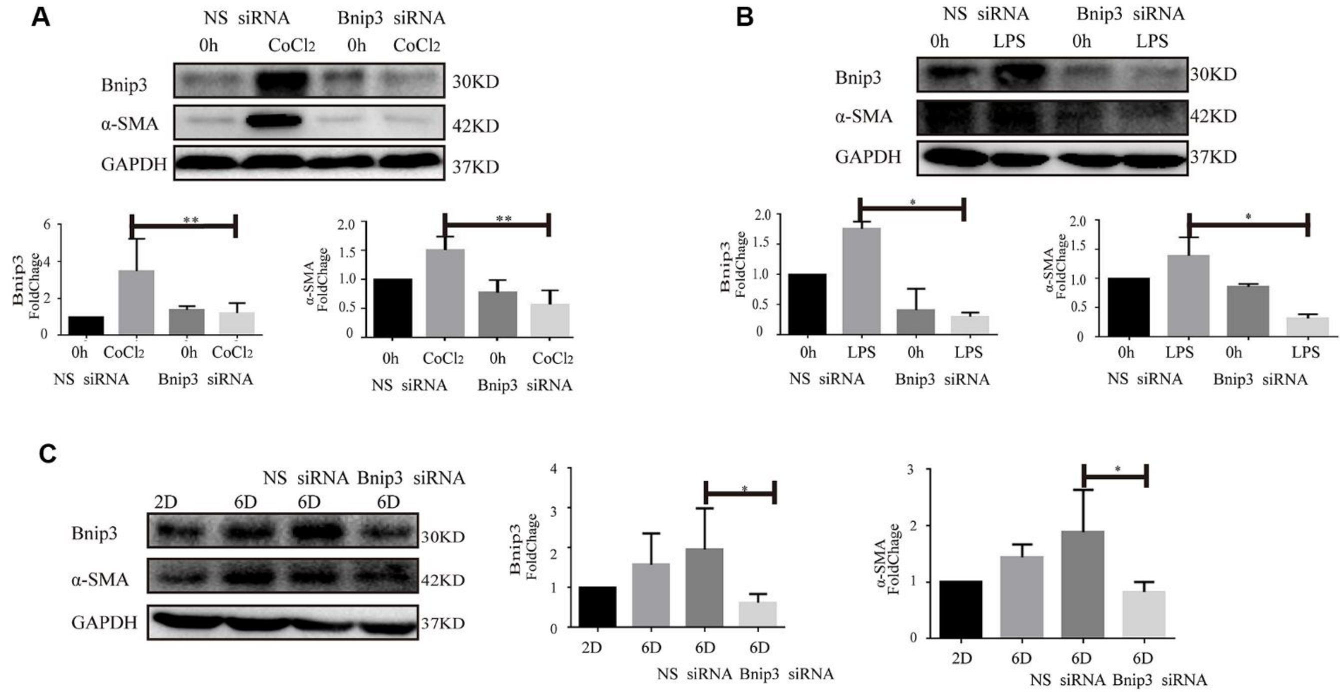

Supplementary Figure 2. Interference of Bnip3 expression inhibited the activation of hepatic stellate cells. LX-2 cells were stimulated by 100 μM $CoCl_2$ (**A**) or 2 μg/ml LPS (**B**), either alone or after *Bnip3* siRNA transfection. Cells were collected at 48 h post transfection and the expression of Bnip3 and α-SMA was detected by Western blot. (**C**) Primary HSCs were isolated from mice and cultivated in vitro. Cells were transfected with specific siRNA targeting *Bnip3* as cells were cultivated up to Day 3 and the expressions of Bnip3 and α-SMA were detected by Western blot. Densitometric analysis was performed and data were expressed as mean ± SD, *P < 0.05, **P < 0.01.

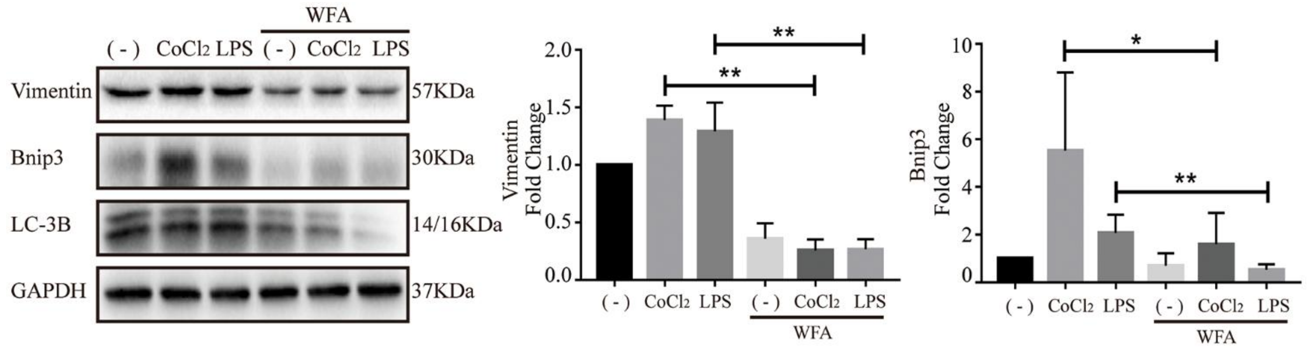

Supplementary Figure 3. Inhibition of vimentin re-organization inhibited Bnip3 expression and autophagy in hypoxia or LPS stimulated LX-2 cells. LX-2 cells were stimulated by 100 μM $CoCl_2$ or 2 μg/ml LPS either alone or after Withaferin A (1.0 μM) pre-treatment and the expression of vimentin, Bnip3 and LC3B was detected by Western blot. Densitometric analysis was performed and data were expressed as mean ± SD, *$P < 0.05$, **$P < 0.01$.
