# Supplementary material for: Bnip3 interacts with vimentin, an intermediate filament protein, and regulates autophagy of hepatic stellate cells
Source: Aging (Albany NY). 2020 Dec 3;13(1):957–72. doi: 10.18632/aging.202211 (PMC7834981; doi:10.18632/aging.202211)
Supplement: Supplementary Table 1 [file aging-13-202211-s002.docx]

**Supplementary Table 1. Screen of proteins interacted with Bnip3 in non-stimulated LX-2 cells with mass spectrometry.**

| **Accession** | **Description** | **Score** | **Coverage** | **# Proteins** | **# Unique Peptides** | **# Peptides** | **# PSMs** | **# AAs** | **MW [kDa]** | **calc. pI** |
| --- | --- | --- | --- | --- | --- | --- | --- | --- | --- | --- |
| P35579 | Myosin-9 OS=Homo sapiens GN=MYH9 PE=1 SV=4 - [MYH9_HUMAN] | 129.42 | 19.59 | 6 | 22 | 30 | 65 | 1960 | 226.4 | 5.60 |
| P60709 | Actin, cytoplasmic 1 OS=Homo sapiens GN=ACTB PE=1 SV=1 - [ACTB_HUMAN] | 115.88 | 44.53 | 21 | 4 | 13 | 55 | 375 | 41.7 | 5.48 |
| P68032 | Actin, alpha cardiac muscle 1 OS=Homo sapiens GN=ACTC1 PE=1 SV=1 - [ACTC_HUMAN] | 66.80 | 29.71 | 14 | 1 | 10 | 40 | 377 | 42.0 | 5.39 |
| P35580 | Myosin-10 OS=Homo sapiens GN=MYH10 PE=1 SV=3 - [MYH10_HUMAN] | 62.86 | 5.57 | 3 | 2 | 10 | 27 | 1976 | 228.9 | 5.54 |
| A0A0C4DGB6 | Serum albumin OS=Homo sapiens GN=ALB PE=1 SV=1 - [A0A0C4DGB6_HUMAN] | 52.29 | 8.61 | 8 | 5 | 5 | 26 | 604 | 69.2 | 6.37 |
| P38646 | Stress-70 protein, mitochondrial OS=Homo sapiens GN=HSPA9 PE=1 SV=2 - [GRP75_HUMAN] | 44.66 | 19.88 | 4 | 10 | 10 | 19 | 679 | 73.6 | 6.16 |
| P68104 | Elongation factor 1-alpha 1 OS=Homo sapiens GN=EEF1A1 PE=1 SV=1 - [EF1A1_HUMAN] | 30.79 | 13.42 | 7 | 6 | 6 | 14 | 462 | 50.1 | 9.01 |
| E9PKE3 | Heat shock cognate 71 kDa protein OS=Homo sapiens GN=HSPA8 PE=1 SV=1 - [E9PKE3_HUMAN] | 25.65 | 11.64 | 19 | 5 | 7 | 13 | 627 | 68.76 | 5.52 |
| P11021 | 78 kDa glucose-regulated protein OS=Homo sapiens GN=HSPA5 PE=1 SV=2 - [GRP78_HUMAN] | 23.50 | 8.87 | 1 | 2 | 4 | 11 | 654 | 72.29 | 5.16 |
| H0YL52 | Tropomyosin alpha-1 chain (Fragment) OS=Homo sapiens GN=TPM1 PE=1 SV=1 - [H0YL52_HUMAN] | 22.48 | 20.38 | 22 | 3 | 5 | 9 | 265 | 30.5 | 4.69 |
| P04279 | Semenogelin-1 OS=Homo sapiens GN=SEMG1 PE=1 SV=2 - [SEMG1_HUMAN] | 18.89 | 14.72 | 2 | 5 | 5 | 9 | 462 | 52.1 | 9.29 |
| F8W1R7 | Myosin light polypeptide 6 OS=Homo sapiens GN=MYL6 PE=1 SV=1 - [F8W1R7_HUMAN] | 17.66 | 35.86 | 12 | 4 | 4 | 8 | 145 | 16.3 | 4.65 |
| A0A087WWU8 | Tropomyosin alpha-3 chain OS=Homo sapiens GN=TPM3 PE=1 SV=1 - [A0A087WWU8_HUMAN] | 16.15 | 19.82 | 10 | 2 | 4 | 8 | 227 | 26.4 | 4.78 |
| P19105 | Myosin regulatory light chain 12A OS=Homo sapiens GN=MYL12A PE=1 SV=2 - [ML12A_HUMAN] | 15.80 | 28.07 | 5 | 4 | 4 | 7 | 171 | 19.8 | 4.81 |
| P62805 | Histone H4 OS=Homo sapiens GN=HIST1H4A PE=1 SV=2 - [H4_HUMAN] | 15.65 | 21.36 | 1 | 2 | 2 | 7 | 103 | 11.4 | 11.36 |
| B0YJC4 | Vimentin OS=Homo sapiens GN=VIM PE=1 SV=1 - [B0YJC4_HUMAN] | 15.07 | 9.05 | 5 | 4 | 4 | 7 | 431 | 49.6 | 5.25 |
| Q5ST81 | Tubulin beta chain OS=Homo sapiens GN=TUBB PE=1 SV=1 - [Q5ST81_HUMAN] | 13.93 | 12.90 | 20 | 4 | 4 | 8 | 372 | 41.7 | 4.91 |
| Q9BQE3 | Tubulin alpha-1C chain OS=Homo sapiens GN=TUBA1C PE=1 SV=1 - [TBA1C_HUMAN] | 9.26 | 9.80 | 20 | 3 | 3 | 4 | 449 | 49.9 | 5.10 |
| P62851 | 40S ribosomal protein S25 OS=Homo sapiens GN=RPS25 PE=1 SV=1 - [RS25_HUMAN] | 6.39 | 7.20 | 1 | 1 | 1 | 3 | 125 | 13.7 | 10.11 |
| E7EUT5 | Glyceraldehyde-3-phosphate dehydrogenase OS=Homo sapiens GN=GAPDH PE=1 SV=1 - [E7EUT5_HUMAN] | 5.57 | 2.69 | 3 | 1 | 1 | 3 | 260 | 27.9 | 6.95 |
| O60814 | Histone H2B type 1-K OS=Homo sapiens GN=HIST1H2BK PE=1 SV=3 - [H2B1K_HUMAN] | 4.48 | 19.05 | 16 | 2 | 2 | 3 | 126 | 13.9 | 10.32 |
| F6RP06 | BCL2/adenovirus E1B 19 kDa protein-interacting protein 3 (Fragment) OS=Homo sapiens GN=BNIP3 PE=1 SV=1 - [F6RP06_HUMAN] | 4.29 | 5.02 | 3 | 1 | 1 | 2 | 219 | 23.71 | 7.20 |
| A0A075B6E2 | 40S ribosomal protein S19 OS=Homo sapiens GN=RPS19 PE=1 SV=1 - [A0A075B6E2_HUMAN] | 4.06 | 12.68 | 2 | 1 | 1 | 2 | 71 | 7.83 | 11.00 |
| F8W026 | Endoplasmin (Fragment) OS=Homo sapiens GN=HSP90B1 PE=1 SV=8 - [F8W026_HUMAN] | 3.97 | 25 | 5 | 1 | 1 | 2 | 48 | 5.48 | 4.89 |
| P19338 | Nucleolin OS=Homo sapiens GN=NCL PE=1 SV=3 - [NUCL_HUMAN] | 3.89 | 1.27 | 1 | 1 | 1 | 2 | 710 | 76.6 | 4.70 |
| J3JS69 | 40S ribosomal protein S18 OS=Homo sapiens GN=RPS18 PE=1 SV=1 - [J3JS69_HUMAN] | 3.85 | 24.39 | 2 | 2 | 2 | 3 | 82 | 9.8 | 11.41 |
| C9J0D1 | Histone H2A OS=Homo sapiens GN=H2AFV PE=1 SV=1 - [C9J0D1_HUMAN] | 3.75 | 7.38 | 18 | 1 | 1 | 2 | 122 | 13.2 | 9.99 |
| M0R3H0 | 40S ribosomal protein S16 OS=Homo sapiens GN=RPS16 PE=1 SV=1 - [M0R3H0_HUMAN] | 2.56 | 11.00 | 4 | 1 | 1 | 1 | 100 | 11.1 | 10.24 |
| K7EJT5 | 60S ribosomal protein L22 (Fragment) OS=Homo sapiens GN=RPL22 PE=1 SV=1 - [K7EJT5_HUMAN] | 2.22 | 27.66 | 7 | 1 | 1 | 1 | 47 | 5.1 | 9.42 |
| P62750 | 60S ribosomal protein L23a OS=Homo sapiens GN=RPL23A PE=1 SV=1 - [RL23A_HUMAN] | 2.22 | 6.41 | 5 | 1 | 1 | 1 | 156 | 17.7 | 10.45 |
| Q96IG2 | F-box/LRR-repeat protein 20 OS=Homo sapiens GN=FBXL20 PE=1 SV=2 - [FXL20_HUMAN] | 2.07 | 4.36 | 2 | 1 | 1 | 4 | 436 | 48.4 | 7.49 |
| P06748 | Nucleophosmin OS=Homo sapiens GN=NPM1 PE=1 SV=2 - [NPM_HUMAN] | 1.96 | 3.06 | 1 | 1 | 1 | 1 | 294 | 32.6 | 4.78 |
| H0YB22 | 40S ribosomal protein S14 (Fragment) OS=Homo sapiens GN=RPS14 PE=1 SV=1 - [H0YB22_HUMAN] | 1.95 | 9.17 | 3 | 1 | 1 | 1 | 120 | 12.9 | 9.85 |
| E9PSF4 | 40S ribosomal protein S3 OS=Homo sapiens GN=RPS3 PE=1 SV=1 - [E9PSF4_HUMAN] | 1.87 | 12.62 | 5 | 1 | 1 | 1 | 103 | 11.2 | 9.31 |
| Q8IYB1 | Protein MB21D2 OS=Homo sapiens GN=MB21D2 PE=1 SV=3 - [M21D2_HUMAN] | 1.74 | 1.83 | 1 | 1 | 1 | 1 | 491 | 55.8 | 7.03 |
| F8VWV4 | 60S acidic ribosomal protein P0 (Fragment) OS=Homo sapiens GN=RPLP0 PE=1 SV=1 - [F8VWV4_HUMAN] | 0.00 | 6.31 | 12 | 1 | 1 | 1 | 111 | 12.2 | 9.25 |
