# Supplementary material for: Bnip3 interacts with vimentin, an intermediate filament protein, and regulates autophagy of hepatic stellate cells
Source: Aging (Albany NY). 2020 Dec 3;13(1):957–72. doi: 10.18632/aging.202211 (PMC7834981; doi:10.18632/aging.202211)
Supplement: Supplementary Table 2 [file aging-13-202211-s003.docx]

Supplementary Table 2. Screen of proteins interacted with Bnip3 in hypoxia-stimulated LX-2 cells with mass spectrometry.

| **Accession** | **Description** | **Score** | **Coverage** | **# Proteins** | **# Unique Peptides** | **# Peptides** | **# PSMs** | **# AAs** | **MW [kDa]** | **calc. pI** |
| --- | --- | --- | --- | --- | --- | --- | --- | --- | --- | --- |
| P38646 | Stress-70 protein, mitochondrial OS=Homo sapiens GN=HSPA9 PE=1 SV=2 - [GRP75_HUMAN] | 127.80 | 34.32 | 5.00 | 18.00 | 18.00 | 60.00 | 679.00 | 73.63 | 6.16 |
| P35579 | Myosin-9 OS=Homo sapiens GN=MYH9 PE=1 SV=4 - [MYH9_HUMAN] | 98.57 | 18.11 | 7.00 | 20.00 | 27.00 | 54.00 | 1960.00 | 226.39 | 5.60 |
| P60709 | Actin, cytoplasmic 1 OS=Homo sapiens GN=ACTB PE=1 SV=1 - [ACTB_HUMAN] | 90.79 | 50.13 | 21.00 | 5.00 | 13.00 | 47.00 | 375.00 | 41.71 | 5.48 |
| P68032 | Actin, alpha cardiac muscle 1 OS=Homo sapiens GN=ACTC1 PE=1 SV=1 - [ACTC_HUMAN] | 63.18 | 27.85 | 14.00 | 1.00 | 9.00 | 34.00 | 377.00 | 41.99 | 5.39 |
| A0A0C4DGB6 | Serum albumin OS=Homo sapiens GN=ALB PE=1 SV=1 - [A0A0C4DGB6_HUMAN] | 49.79 | 9.77 | 8.00 | 6.00 | 6.00 | 23.00 | 604.00 | 69.18 | 6.37 |
| P11142 | Heat shock cognate 71 kDa protein OS=Homo sapiens GN=HSPA8 PE=1 SV=1 - [HSP7C_HUMAN] | 47.40 | 24.15 | 19.00 | 11.00 | 13.00 | 28.00 | 646.00 | 70.85 | 5.52 |
| P11021 | 78 kDa glucose-regulated protein OS=Homo sapiens GN=HSPA5 PE=1 SV=2 - [GRP78_HUMAN] | 40.18 | 15.90 | 3.00 | 6.00 | 8.00 | 21.00 | 654.00 | 72.29 | 5.16 |
| P35580 | Myosin-10 OS=Homo sapiens GN=MYH10 PE=1 SV=3 - [MYH10_HUMAN] | 36.44 | 4.40 | 3.00 | 1.00 | 8.00 | 20.00 | 1976.00 | 228.86 | 5.54 |
| P62805 | Histone H4 OS=Homo sapiens GN=HIST1H4A PE=1 SV=2 - [H4_HUMAN] | 26.76 | 40.78 | 1.00 | 4.00 | 4.00 | 12.00 | 103.00 | 11.36 | 11.36 |
| B0YJC4 | Vimentin OS=Homo sapiens GN=VIM PE=1 SV=1 - [B0YJC4_HUMAN] | 21.08 | 9.74 | 5.00 | 4.00 | 4.00 | 9.00 | 431.00 | 49.62 | 5.25 |
| O60814 | Histone H2B type 1-K OS=Homo sapiens GN=HIST1H2BK PE=1 SV=3 - [H2B1K_HUMAN] | 19.85 | 19.05 | 16.00 | 2.00 | 2.00 | 10.00 | 126.00 | 13.88 | 10.32 |
| F8W1R7 | Myosin light polypeptide 6 OS=Homo sapiens GN=MYL6 PE=1 SV=1 - [F8W1R7_HUMAN] | 15.18 | 25.52 | 12.00 | 3.00 | 3.00 | 7.00 | 145.00 | 16.28 | 4.65 |
| P19105 | Myosin regulatory light chain 12A OS=Homo sapiens GN=MYL12A PE=1 SV=2 - [ML12A_HUMAN] | 14.45 | 29.82 | 5.00 | 5.00 | 5.00 | 7.00 | 171.00 | 19.78 | 4.81 |
| F6RP06 | BCL2/adenovirus E1B 19 kDa protein-interacting protein 3 (Fragment) OS=Homo sapiens GN=BNIP3 PE=1 SV=1 - [F6RP06_HUMAN] | 14.34 | 11.87 | 3.00 | 3.00 | 3.00 | 7.00 | 219.00 | 23.71 | 7.20 |
| P68363 | Tubulin alpha-1B chain OS=Homo sapiens GN=TUBA1B PE=1 SV=1 - [TBA1B_HUMAN] | 12.20 | 14.41 | 18.00 | 5.00 | 5.00 | 8.00 | 451.00 | 50.12 | 5.06 |
| A0A087WVQ9 | Elongation factor 1-alpha 1 OS=Homo sapiens GN=EEF1A1 PE=1 SV=1 - [A0A087WVQ9_HUMAN] | 11.87 | 15.65 | 7.00 | 5.00 | 5.00 | 7.00 | 441.00 | 47.85 | 9.03 |
| P62263 | 40S ribosomal protein S14 OS=Homo sapiens GN=RPS14 PE=1 SV=3 - [RS14_HUMAN] | 9.46 | 15.89 | 3.00 | 2.00 | 2.00 | 4.00 | 151.00 | 16.26 | 10.05 |
| Q5ST81 | Tubulin beta chain OS=Homo sapiens GN=TUBB PE=1 SV=1 - [Q5ST81_HUMAN] | 9.41 | 17.74 | 22.00 | 4.00 | 4.00 | 5.00 | 372.00 | 41.72 | 4.91 |
| J3JS69 | 40S ribosomal protein S18 OS=Homo sapiens GN=RPS18 PE=1 SV=1 - [J3JS69_HUMAN] | 7.24 | 24.39 | 2.00 | 2.00 | 2.00 | 5.00 | 82.00 | 9.76 | 11.41 |
| Q5JVD1 | Centriolin OS=Homo sapiens GN=CNTRL PE=1 SV=1 - [Q5JVD1_HUMAN] | 5.45 | 1.01 | 2.00 | 1.00 | 1.00 | 3.00 | 994.00 | 113.69 | 5.21 |
| H0YKX5 | Tropomyosin alpha-1 chain (Fragment) OS=Homo sapiens GN=TPM1 PE=1 SV=1 - [H0YKX5_HUMAN] | 4.81 | 16.20 | 20.00 | 1.00 | 2.00 | 4.00 | 142.00 | 16.36 | 4.74 |
| P39019 | 40S ribosomal protein S19 OS=Homo sapiens GN=RPS19 PE=1 SV=2 - [RS19_HUMAN] | 4.42 | 15.17 | 6.00 | 2.00 | 2.00 | 2.00 | 145.00 | 16.05 | 10.32 |
| Q8IYB1 | Protein MB21D2 OS=Homo sapiens GN=MB21D2 PE=1 SV=3 - [M21D2_HUMAN] | 4.16 | 4.28 | 1.00 | 2.00 | 2.00 | 2.00 | 491.00 | 55.76 | 7.03 |
| P30050 | 60S ribosomal protein L12 OS=Homo sapiens GN=RPL12 PE=1 SV=1 - [RL12_HUMAN] | 4.03 | 5.45 | 1.00 | 1.00 | 1.00 | 2.00 | 165.00 | 17.81 | 9.42 |
| C9J0D1 | Histone H2A OS=Homo sapiens GN=H2AFV PE=1 SV=1 - [C9J0D1_HUMAN] | 3.87 | 13.11 | 20.00 | 2.00 | 2.00 | 2.00 | 122.00 | 13.16 | 9.99 |
| H0YBC7 | BCL2/adenovirus E1B 19 kDa protein-interacting protein 3-like (Fragment) OS=Homo sapiens GN=BNIP3L PE=1 SV=1 - [H0YBC7_HUMAN] | 3.57 | 8.11 | 2.00 | 1.00 | 1.00 | 3.00 | 185.00 | 20.08 | 5.71 |
| V9GYG0 | ADP/ATP translocase 1 OS=Homo sapiens GN=SLC25A4 PE=1 SV=1 - [V9GYG0_HUMAN] | 2.34 | 5.77 | 5.00 | 1.00 | 1.00 | 1.00 | 208.00 | 22.87 | 9.54 |
| A0A0C4DGC5 | Prelamin-A/C (Fragment) OS=Homo sapiens GN=LMNA PE=1 SV=1 - [A0A0C4DGC5_HUMAN] | 2.33 | 4.23 | 3.00 | 1.00 | 1.00 | 1.00 | 260.00 | 27.52 | 9.69 |
| K7EMV3 | Histone H3 OS=Homo sapiens GN=H3F3B PE=1 SV=1 - [K7EMV3_HUMAN] | 2.25 | 9.78 | 11.00 | 1.00 | 1.00 | 1.00 | 92.00 | 10.33 | 11.82 |
| H0Y8D1 | Trypsin-1 (Fragment) OS=Homo sapiens GN=PRSS1 PE=1 SV=1 - [H0Y8D1_HUMAN] | 2.07 | 7.04 | 4.00 | 1.00 | 1.00 | 2.00 | 142.00 | 15.41 | 7.27 |
| P06748 | Nucleophosmin OS=Homo sapiens GN=NPM1 PE=1 SV=2 - [NPM_HUMAN] | 2.02 | 10.88 | 2.00 | 2.00 | 2.00 | 2.00 | 294.00 | 32.55 | 4.78 |
| P46783 | 40S ribosomal protein S10 OS=Homo sapiens GN=RPS10 PE=1 SV=1 - [RS10_HUMAN] | 2.00 | 5.45 | 3.00 | 1.00 | 1.00 | 2.00 | 165.00 | 18.89 | 10.15 |
| K7EJT5 | 60S ribosomal protein L22 (Fragment) OS=Homo sapiens GN=RPL22 PE=1 SV=1 - [K7EJT5_HUMAN] | 1.99 | 27.66 | 7.00 | 1.00 | 1.00 | 1.00 | 47.00 | 5.08 | 9.42 |
| Q7Z478 | ATP-dependent RNA helicase DHX29 OS=Homo sapiens GN=DHX29 PE=1 SV=2 - [DHX29_HUMAN] | 1.95 | 0.66 | 2.00 | 1.00 | 1.00 | 1.00 | 1369.00 | 155.14 | 8.09 |
| E7EUT5 | Glyceraldehyde-3-phosphate dehydrogenase OS=Homo sapiens GN=GAPDH PE=1 SV=1 - [E7EUT5_HUMAN] | 1.93 | 2.69 | 3.00 | 1.00 | 1.00 | 1.00 | 260.00 | 27.85 | 6.95 |
| A0A087WUI2 | Heterogeneous nuclear ribonucleoproteins A2/B1 OS=Homo sapiens GN=HNRNPA2B1 PE=1 SV=1 - [A0A087WUI2_HUMAN] | 1.82 | 5.75 | 10.00 | 2.00 | 2.00 | 2.00 | 261.00 | 29.82 | 4.97 |
| H0YAP2 | Polyadenylate-binding protein 1 (Fragment) OS=Homo sapiens GN=PABPC1 PE=1 SV=1 - [H0YAP2_HUMAN] | 1.69 | 6.11 | 7.00 | 1.00 | 1.00 | 1.00 | 131.00 | 15.15 | 9.64 |
| Q9UKJ8 | Disintegrin and metalloproteinase domain-containing protein 21 OS=Homo sapiens GN=ADAM21 PE=2 SV=2 - [ADA21_HUMAN] | 0.00 | 2.35 | 1.00 | 1.00 | 1.00 | 1.00 | 722.00 | 80.78 | 6.68 |
| Q9Y442 | Uncharacterized protein C22orf24 OS=Homo sapiens GN=C22orf24 PE=2 SV=1 - [CV024_HUMAN] | 0.00 | 7.50 | 1.00 | 1.00 | 1.00 | 1.00 | 160.00 | 17.72 | 8.15 |
| Q96IG2 | F-box/LRR-repeat protein 20 OS=Homo sapiens GN=FBXL20 PE=1 SV=2 - [FXL20_HUMAN] | 0.00 | 4.36 | 2.00 | 1.00 | 1.00 | 1.00 | 436.00 | 48.39 | 7.49 |
| Q14568 | Heat shock protein HSP 90-alpha A2 OS=Homo sapiens GN=HSP90AA2P PE=1 SV=2 - [HS902_HUMAN] | 0.00 | 2.62 | 4.00 | 1.00 | 1.00 | 2.00 | 343.00 | 39.34 | 4.65 |
| D6R904 | Tropomyosin alpha-3 chain OS=Homo sapiens GN=TPM3 PE=1 SV=1 - [D6R904_HUMAN] | 0.00 | 25.26 | 17.00 | 1.00 | 2.00 | 2.00 | 95.00 | 11.01 | 4.79 |
| M0QX76 | 40S ribosomal protein S16 (Fragment) OS=Homo sapiens GN=RPS16 PE=1 SV=1 - [M0QX76_HUMAN] | 0.00 | 20.00 | 6.00 | 1.00 | 1.00 | 1.00 | 50.00 | 5.55 | 9.63 |
| E9PRF4 | Histone-lysine N-methyltransferase (Fragment) OS=Homo sapiens GN=SETDB1 PE=1 SV=1 - [E9PRF4_HUMAN] | 0.00 | 2.62 | 2.00 | 1.00 | 1.00 | 1.00 | 1259.00 | 139.30 | 6.21 |
| H0YNM2 | Semaphorin-4B (Fragment) OS=Homo sapiens GN=SEMA4B PE=1 SV=1 - [H0YNM2_HUMAN] | 0.00 | 5.00 | 1.00 | 1.00 | 1.00 | 1.00 | 120.00 | 13.06 | 7.99 |
| A0A075B6Z2 | T-cell receptor alpha joining 56 (Fragment) OS=Homo sapiens GN=TRAJ56 PE=4 SV=1 - [A0A075B6Z2_HUMAN] | 0.00 | 38.10 | 1.00 | 1.00 | 1.00 | 8.00 | 21.00 | 2.22 | 10.29 |
| B7ZBA8 | Coiled-coil domain-containing protein 154 OS=Homo sapiens GN=CCDC154 PE=1 SV=1 - [B7ZBA8_HUMAN] | 0.00 | 1.15 | 2.00 | 1.00 | 1.00 | 1.00 | 522.00 | 59.41 | 9.26 |
| A0A0G2JNN3 | Leukocyte receptor cluster member 8 (Fragment) OS=Homo sapiens GN=LENG8 PE=1 SV=1 - [A0A0G2JNN3_HUMAN] | 0.00 | 1.24 | 5.00 | 1.00 | 1.00 | 1.00 | 726.00 | 79.44 | 9.55 |
